# Some Points in the Natural History of Caries of the Teeth, and the Value of Fillings for Its Arrest

**Published:** 1880-12

**Authors:** G. V. Black

**Affiliations:** Jacksonville, Ill.


### THE DENTAL REGISTER.

Vol. XXXIV.] DECEMBER, 1880. [No. 12.

### SOME POINTS IN THE NATURAL HISTORY OF CARIES OF THE TEETH, AND THE VALUE OF FILLINGS FOR ITS ARREST.

By G. V. Black, D. D. S., of Jacksonville, Ill.

Perhaps the best way to estimate the value of fillings, is to study the results in connection with an extended study of the habits and tendencies of caries of the teeth when allowed to pursue its course. The best results in the study of medicine within the last half century, have been obtained by the close observations made on disease, which has been allowed to pursue its course without therapeutic interference. By this means many errors of opinion respecting the tendency of individual diseases have been corrected, and a better knowledge of them arrived at. Indeed it is now recognized that in order to know a disease well, it must be studied without therapeutic interference—by no other means can we so certainly arrive at its true nature, habits and tendencies. Having thus gained an understanding of the disease, we are ready to note understandingly the modifying influence of drugs—not before—for certainly we can not be supposed to understand what good or evil a drug has done in changing the course of a disease, if we have no real knowledge of the course of the disease when not modified by drugs.

Each disease has its habits and tendencies, which are more or less fixed, and each case pursues a course very similar to each other case of like nature or type. Each specific disease also arises from a specific cause; always from that one cause, and from no other, as certainly as an oak arises from an acorn, or a bird from an egg. In this catalogue of specific diseases, we, of course, do not include those accidental diseases which run no specific course, and probably arise from causes purely accidental. Yet it is well understood that the progress of discovery is continually transferring important lessons to the list of specific diseases.

Decay of the teeth is certainly a specific disease, running a specific course, and evidently arises from a certain specific cause, but this cause is not as yet certainly known. It may be regarded as a malignant disease, in that its general tendency is to the entire destruction of the organ attacked. This malignancy, however, receives a decided check in the fact that there is absolute immunity for all the other organs of the body than the teeth, hence the disease is always strictly localized. Whether its specific cause be local or general, is not yet certainly known. Not being able to trace the cause, our only knowledge of the disease is its phenomena, and it is to these phenomena that we now wish to direct attention. In this paper we can only glance at a few points. It was no part of the plan of this paper to enter into a discussion of the *cause* of decay, but I may be pardoned for introducing a few propositions on that subject, which will in some measure represent my conception of the facts now known to me.

1. There is no decay without an acid condition of the fluids of the mouth.

2. If acidity alone be the cause of decay it must be due to state or condition of the acid not yet known or recognized.

3. While an acid is not only always present, but is probably a necessity to the inception and progress of decay, there may be an agent acting in conjunction with the acid that is not yet known or recognized.

4. While there is no decay without the presence of an acid, there is not necessarily decay because of the presence of an acid.

5. Decay is in no wise dependent upon the physiological perfection, or imperfection of tooth structure any farther than that imperfections of the surface of a tooth may afford favorable points for the beginnings of decay.

6. The force of vitality has no influence in the prevention of decay. But in living dentine decay progresses less rapidly, probably because the tubules being pefectly filled with the fibrils, tends to prevent the ingress of the materies morbi, and in this manner limits the surface upon which it may exert its influence, and more rapidly in soft teeth than in hard ones, because there is less basic substance to be broken down.

My object in experimenting with acids in motion, of which a partial report was made to the American Dental Association, also to the Mississippi Valley Dental Association, was to see if this change in the condition of the acid would produce a change in its action. My observations had led me to the conclusion that certain changes would be produced. The result of the experiments, while they demonstrated a change of action, showed a different change from that anticipated be me before beginning. All my efforts to detect a special agent acting jointly with an acid for the production of decay, have so far entirely failed. I have never been able to satisfy myself, either by experiment, observation, or otherwise, that *Leptothrix Buccalis* or other fungi demonstrable with the microscope acted as such morbific agent, or stood in the relation of causation to decay of the teeth.

We will now leave the causes of decay entirely, and in the rest of this paper we will regard it simply as the result of a force, without reference to the cause or origin of this force.

Decay is disintegration of the tooth substance, molecule by molecule. It always begins on the surface of the tooth and slowly makes its way toward the central parts, spreading as it proceeds. The dentine is destroyed more rapidly than the enamel so that in most instances the dentine is burrowed out beneath the enamel in such manner that the diameter of the cavity just within is larger than the opening.

Decay has pretty definitely fixed habits as to the point upon a tooth that is attacked, and one of the most notable characteristics is that it never attacks a tooth upon a surface that is smooth and is constantly worn or is kept clean by the attrition of mastication, or the friction of the lips, cheeks or tongue. All such points are absolutely exempt from attack.

The points of attack may be divided into four classes:

1. Pits and groves in the enamel.
2. Approximal surfaces.

3. Points which are from any cause habitually unclean.

4, At the junction of the cementum and enamel.

The *first class* is usually the first to make its appearance. It occurs in the pits and grooves of the enamel wherever found. In the molars, in the pits and grooves of the grinding surface, and in the groove which is often present in the buccal surface In the bicuspids, in the pits and grooves of the grinding surface. In the incisors, in the pit often present in the lingual surface. In pits and grooves, the result of faulty formation in any of the teeth.

The *second class* occurs in the approximal surfaces of any or all of the teeth below the junction of the cementum with the enamel, and is usually the second to make its appearance.

The *third class* rarely makes its appearance except in uncleanly mouths, or among those who habitually sleep with the mouth open. Their position is the smooth surfaces—usually the buccal or labial—of any or of all of the teeth below the junction of the cementum with the enamel.

The *fourth class* is always accompanied with diseased gums, which has usually caused more or less exposure of the cementum, and has its beginning just at the joining of the cementum and enamel. It is most generally seen in the middle or in old age. very rarely in children or young persons.

The first two classes belong to youth or early maturity. If they are not seen until later it is on account of their slow progress. After watching this point critically, I have become satisfied that very few decays in these positions originate after the age of twenty-five years; and as these comprise about ninety per cent. of all decay, we may conclude that caries of the teeth, in its inception at least, is a disease of youth.

We wish now to call especial attention to two points. We all know that mouths are frequently met with in which there are many decays, which at middle life have not progressed so far as to do material injury. Many points have been attacked, but the destructive process has been extremely slow. Again we often find dentures in which very few decays have started, but these few have progressed with great rapidity, and several teeth have been lost, while perhaps all, or nearly all, of the remaning ones are free from decay. There are the following variations:

1. Cases in which *few* decays start and the destructive process is *slow.*

2. Cases in which *many* decays start and the destructive process is *slow.*

3, Cases occur where few decays start and the destructive process is rapid.

4. Cases occur where many decays start and the destructive process is rapid.

To represent these characteristics, we propose the terms "vis inita," beginning-power—from *vis*, power, force; and *ineo*, to begin—and "vis deleta," destroying-power—from *deleo*, to blot out, to destroy. With these words representing the quality of the power of caries, we may associate numbers to represent our conception of the degree of that power in a given case.

The following are the extremes of possible combinations:

Vis inita 1, with vis deleta 1 to 100.

Vis inita 100, with vis deleta 1 to 100.

For some years past we have made a special study of some of these characteristics of decay, particularly those of which we now speak, and we are satisfied that a more close and thoughtful study of them will be of very great benefit, both to operator and patient, and at the same time serve to correct many misapprehensions as to the real value of fillings. And we have thought that the selection of some simple words not in common use, which might be clothed with a specific meaning to represent them, would not only be acceptable but helpful.

Now, as to the character of these powers, we do not for a moment wish to be understood as attributing the beginning of decay, the vis inita, and the destructive power, vis deleta, to different causes, but should rather say that these are manifestations of one force, but of different qualities of that force, or manifestations under differences of environment. In the one case the action is on the surface of the tooth, or in the shallow pit or groove, where the general fluids of the mouth have tolerably free access to the part or tissue being acted upon, while the other is much more secluded from disturbing influences—is deeper in the tooth—more hidden away. Again, the vis inita is exerted for the most part upon enamel, while the vis deleta is spent mostly upon the dentine, or with our present lack of knowledge of the cause of decay, it might be regarded as an arbitrary division for the study of phenomena. Whatever may be the cause of these differences, it is clear enough that they exist and modify powerfully the conditions under which we are called upon for dental operations, and exert a great influence upon the value or protective power of fillings.

It is not uncommon to find persons who have had no dental operations whatever, with some teeth missing, or only roots remaining here and there through the mouth. An examination proves that most of the other teeth are free from decay. The teeth that are wanting have been attacked and quickly destroyed by decay, while the remaining ones have not been attacked at all. In this case we would say, perhaps, that there was vis inita, 25; vis deleta, 90

In another case we may find the denture of a patient at middle life with many small, dark cavities, none of which have progressed so far as to give any trouble. For the practical purposes of mastication the teeth are as yet uninjured. Only a short time ago I examined such a case, in which I should say there was *vis inita*, 80; vis deleta, 10. Many decays had started in the teeth, but that power which drives on to quick and sure destruction which we so often see to our sorrow, was wanting.

It was very different with a pair of twins, eighteen years old Swedish girls, who came to me for advice, last October. In the mouths of these girls there was not a tooth, except the wisdom teeth (which were not yet through the gums), that were not attack at from one to four points; not a tooth which was not from one-half to three-fourths, destroyed by caries; in nearly every tooth there was a living exposed pulp. This, as being the most complete case of destruction I have ever met with, I should place vis inita, 100; vis deleta 100, as the proper expression of the activity of the forces there in operation.

We need not continue the recitation of these cases farther at present, as we think our meaning will be readily understood ; but will now turn our attention to the modifying influence of age, or the different expressions of these powers usually found at different periods of life.

Decay of the teeth is essentially a disease of youth. Especially is this the case with the first and second classes of decay—those starting in the pits and grooves and on the approximal surfaces of the teeth, and to a somewhat lesser extent with the third class. With these, the first and second classes, the vis inita is most strongly manifested before the age of twenty years, and grows less and less active after that age ; and perhaps ceases altogether as early as the fortieth year, except in occasional cases. As the first two classes include most of the cavities met with, we find that if the decays occurring up to this time are successfully protected by fillings, the work of the dentist is about accomplished.

With the vis deleta the case is very different—this may continue for much a longer period. It is less modified by age. We well remember a case which came under our care fifteen years ago. We all remember such cases. The patient was a maiden lady, twenty-eight years old. The vis inita had been very strong as was evident from the fact that almost every tooth remaining in the mouth—five had been lost—had been attacked at from one to three points, except the lower incisors. The vis deleta was less strong, say fifty, but by this time had made sad havoc, as the previous attempts at filling had not been very effectual. The patient wish to have the teeth out and try artificial ones. We protested and made fillings instead. Since that time no new cavity has occurred. Eight years afterwards she was married and has borne one child. During this pregnancy two of the cavities, the fillings in which had been observed to be slightly leaky at the margins, flamed out into rapid decay (vis deleta strong). These points were attended to and no decay has occurred to this day. (These were not plastic fillings.) In this case the vis inita had become almost or quite nothing, with possibly a deminishing of the destructive force, and the remaining vis deleta was mastered by reasonably good fillings until an increase of this force, during pregnancy, searched out and exposed the weak points. Three younger sisters of this patient came under my care at the same time. For them I made fillings, but no tooth has been lost, and for the last five years no filling has been needed.

We might cite very many cases to show that the beginning power of decay is exerted mostly in youth, but deem it unnecessary. The time at which teeth are attacked varies very much in different cases, depending seemingly upon three conditions.

The first is the vis inita, second the physical perfection of the surface of the enamel; third, habitual cleanliness. It will be readily seen that teeth with deep pits and fissures will be attacked in the presence of a weak or moderate vis inita, when teeth of greater physical perfection would escape attack altogether. The same is true of teeth habitually unclean as compared with those that are clean. Both of these conditions vary the manifestations of the vis inita powerfully, favoring it in the one case and disfavoring it in the other. Therefore we have these three considerations before us in the study of the beginnings of decay. If the vis inita is naught we will have no decay. We must have a power or force acting to initiate the process. Mere pits, grooves, or other faulty formation, nor even uncleanness will induce it, unless, indeed, uncleanness carries with it the meteries morbi. While if the vis inita be present, these circumstances will favor or disfavor its manifestation, and if the teeth be unequal in surface perfection, or habitual cleanliness, the expression of the vis inita will be unequal.

As we have said, this force is manifested mostly in youth, and when it is strong the teeth are attacked usually at about a certain time after coming through the gums. That is, if the first molar is found with a decay in its grinding surface two years after coming through the gums, or at eight years, the second molar is likely to have a similar decay when about the same age, or when the patient is fourteen years old. If the incisors have approximal decays five years after coming through, the bicuspids and first molar likely to have approximal cavities at about the same time after they present themselves, or possibly the first molar may be attacked while approximating with the second deciduous molar, and so on with the other teeth in their turn.

Thus we see why it is that we have new cavities occurring continually between the ages of ten and twenty especially, and sometimes continuing until later in life, when the vis inita is less pronounced or more fluctuating. Of course we only present this as the most general rule to which the exceptions are sufficiently numerous. The modifying influences of surface perfection and cleanliness have their weight, which is often very powerful, in modifying the results, and if these are unequal among the different teeth, the manifestations will be unequal. After these come changes of condition as to health with their modifications; conditions which we need not now discuss, as we have considered them at length in a former paper. We will also find that the vis inita often begins to diminish very early in life, so as to lengthen the time between the occurrence of cavities in the different teeth.

Running all through our cases, however, there are many exceptions and exemptions. For instance, the lower incisors are exempt from decay in a very large majority of cases. This is so very decided that we not unfrequently find them sound when all the other teeth have been destroyed. Again, it may happen that some other class of teeth are especially exempt, as the superior incisors, or the canines—occassionaly the bicuspids—but very rarely the molars The wisdom teeth, too, often decay much earlier proportionately, than the other teeth, probably from some peculiarities of environment. All this tends to cover up and conceal the laws of which we have spoken. But when we come to compare a larger number of examinations, or better still, to follow a large number of patients from childhood to middle age, the general rule stands out clearly and unmistakably.

The vis deleta—destroying power—of caries is also subject to various local modifying influences. It is especially and powerfully modified by the condition of the cavity as to its opening, progressing far more rapidly if the opening be small than if it be large, so as to give free ingress and egress to the fluids of the mouth. Indeed if the vis deleta be rather weak, decay, which is moderately progressive in a cavity, the opening of which is small, or partially closed, will cease altogether, if it be thrown wide open to the fluids of the mouth. Just here there is a very curious phenomenon, which

I have studied with much interest. Decay seems to be like the miner cutting coal. To work to advantage he must have a certain amount of room in which to handle his pick. If you cramp him he will go very slowly until he gains room for himself, then he will proceed rapidly with his work. It it is just so with decay. While it is true that to progress most rapidly, the opening of the cavity should be partially closed, a moderately close filling of the cavity will in'erfere with progress for a time, until elbow room--if I may use such an expression—is gained.

It is for this reason that leaky fillings seem to protect for a time, especially in those cases in which the vis deleta is rather weak. However, if the vis deleta be strong, say 75, elbow room (figuratively) is gained so quickly that the decay seems to go right on without check.

Many noticing this in cases of very mild vis deleta, have come to the very erroneous conclusion that it is not necessary that fillings be water tight.

The changes in the destructive power consequent upon the breaking away of the wall of the cavity, and thus throwing it wide open to the fluids of the mouth, and still better to the attrition of mastication, we have seen exemplified in many ways. I was particularly struck with this last summer in my examination of the old Indian remains on the Mississippi. I spent some few weeks in the work, and the results developed some curious facts not before known to me. One of these was the greater prevalence of decay among them than I had previously supposed. The second was the peculiar character of the decay. In a large majority of cases it was confined to the second class, nothing found but approximal decays. A very large proportion of these progressed until the over-laying enamel gave way, and then the hard food wedging into this broke out one side thus exposing the cavity to the attrition of mastication, which, with them, was sufficient to keep it clean and the decay came to a full stop leaving the teeth practically uninjured.

I did, however, find some dentures that had been practically destroyed. One, especially, of a young woman--the wisdom teeth just coming through--in whom the teeth were nearly all destroyed.

There were also evidences of very severe alveolar abscesses which had made extensive burrows in the bony tissues.

This Indian girl seems to have been especially unfortunate, as she had had one femur and both bones of the forearm broken. The femur had healed with two inches shortening, and the arm with about one inch, and quite crooked. I also found a child six years old, with the temporary teeth very badly decayed.

The peculiarities above mentioned, of decay among the Indians, are sometimes seen among our own people. Decays are only found on the approximal surfaces, and the vis deleta being very weak the cavities are broken into before exposure of the pulp takes place and the decay comes to a full stop. The teeth are thus preserved through these natural and unaided processes. Such cases have occosionally come under my observation, as they doubtless have of most dentists of long experience.

Decays arising from want of cleanliness vary greatly as to time of life, but I am convinced that they occur mostly in youth or early maturity. How many decays of the first and second classes are really due to this cause is, of course, very difficult to estimate. I therefore include in this class only those that occur on the smooth and exposed surfaces of the teeth. They are seen mostly on the labial surfaces of the incisors and the buccal surfaces of the bicuspids and molars below the margin of the gum, always below the junction of the cement with the enamel. I think that I have sufficiently demonstrated the dependence of these decays upon uncleannesss, or a local modification of the mucus, in my repeated experiments. It is well known that in these cases the tendency is for tooth after tooth to be attacked in succession, spreading either way from the point of beginning (of course I do not allude to decays starting in congenital or other pits in these surfaces). I have uniformly found that the daily use of the friction of the tooth-brush would prevent more teeth being attacked in this way.

We have yet to notice the fourth-class of decay—those that occur at the margin of the enamel, or at the junction of the cement and enamel. These are peculiar to middle life and advanced age, occurring but rarely in young persons. We believe that the immediate local conditions of their occurrence are to be found in disease of the margins of the gums, and perhaps the margins of the alveolar processes. This is marked by a thickened and irritated condition of the gums at the point, and usually some shrinkage away from the tooth. Some years ago I was inclined to the opinion that this condition was caused by the sharp edges of the cavity, but longer and closer observation has fully convinced me that the disease of the gum is first present, and the decay follows. These decays are not confined to any particular side of the tooth, but occur on the approximal as well as on the buccal and labial surfaces. Such decays also occur frequently where a partial plate abuts against the teeth, causing continued irritation of the margin of the gums. They sometimes progress rapidly and are very destructive. tooth after tooth falling a prey to their ravages. It occasionally happens that after the danger from the first and second classes of decay has passed, this fourth class springs into existence and wrecks the whole denture. We have lost the battle in several of these cases; in others, we seem for the time to have won the fight, and we have now under our care, several cases in which we are several times per year filling decays of this class.--*Trans. of the Illinois State Dental Society.*

